# Does HAART Efficacy Translate to Effectiveness? Evidence for a Trial Effect

**DOI:** 10.1371/journal.pone.0021824

**Published:** 2011-07-13

**Authors:** Prema Menezes, William C. Miller, David A. Wohl, Adaora A. Adimora, Peter A. Leone, William C. Miller, Joseph J. Eron

**Affiliations:** 1 Division of Infectious Diseases, School of Medicine, University of North Carolina at Chapel Hill, Chapel Hill, North Carolina, United States of America; 2 Department of Epidemiology, School of Public Health, University of North Carolina at Chapel Hill, Chapel Hill, North Carolina, United States of America; University of Cape Town, South Africa

## Abstract

**Background:**

Patients who participate in clinical trials may experience better clinical outcomes than patients who initiate similar therapy within clinical care (trial effect), but no published studies have evaluated a trial effect in HIV clinical trials.

**Methods:**

To examine a trial effect we compared virologic suppression (VS) among patients who initiated HAART in a clinical trial versus in routine clinical care. VS was defined as a plasma HIV RNA ≤400 copies/ml at six months after HAART initiation and was assessed within strata of early (1996–99) or current (2000–06) HAART periods. Risk ratios (RR) were estimated using binomial models.

**Results:**

Of 738 persons initiating HAART, 30.6% were women, 61.7% were black, 30% initiated therapy in a clinical trial and 67% (n = 496) had an evaluable six month HIV RNA result. HAART regimens differed between the early and current periods (p<0.001); unboosted PI regimens (55.6%) were more common in the early and NNRTI regimens (46.4%) were more common in the current period. Overall, 78% (95%CI 74, 82%) of patients achieved VS and trial participants were 16% more likely to achieve VS (unadjusted RR 1.16, 95%CI 1.06, 1.27). Comparing trial to non-trial participants, VS differed by study period. In the early period, trial participants initiating HAART were significantly more likely to achieve VS than non-trial participants (adjusted RR 1.33; 95%CI 1.15, 1.54), but not in the current period (adjusted RR 0.98; 95%CI 0.87, 1.11).

**Conclusions:**

A clear clinical trial effect on suppression of HIV replication was observed in the early HAART period but not in the current period.

## Introduction

A trial effect occurs when study participants experience a benefit merely by the act of trial participation. The effect may arise due to a treatment effect (newer, better or experimental treatments available to trial participants but unavailable outside the trial), a protocol effect (differences in the way treatment regimens are delivered), a care effect (differences in care), a Hawthorne effect (behavior change secondary to being under observation) or a placebo effect (“psychologically mediated” benefits that arise due to being in a trial) [1]–[4]. A trial effect should be distinguished from apparent effects (biases) particularly selection bias (differences between trial and non-trial participants).

The evidence for or against a trial participation benefit or trial effect is inconclusive [1], [3], [5]–[7]. Current evidence, derived primarily from cancer trials, is limited in breadth, quality and quantity. HIV-related clinical trials provide an excellent substrate for the measurement of a trial effect. HIV infection is a chronic illness with well-characterized treatments and HIV-related outcomes are easily measured and clinically meaningful.

To determine whether a trial effect exists in HIV clinical trials, we compared virologic suppression (VS) between HIV-infected patients who were antiretroviral naïve and who initiated highly active antiretroviral therapy (HAART) either in a trial or as part of routine medical care. The benefit of HAART to patients is unquestionable. However, if participation in clinical trials leads to a beneficial trial effect, careful consideration of the mechanisms and consequences of that trial effect would be needed. At the least, aspects of the trial effect, such as protocol effect or care effect, may need to be incorporated into clinical care to achieve similar results. Furthermore, the existence of a positive trial effect might suggest reduced generalizability of clinical trials data to non-trial participants. Finally, clinical trials data provide evidence for the care and treatment guidelines of HIV infected persons and a trial effect might oblige guidelines to caution about possible differences in outcome in non-trial settings.

## Methods

### Study design

We conducted a secondary data analysis using the University of North Carolina (UNC) Center for AIDS Research (CFAR) HIV/AIDS clinical cohort (UCHCC). This cohort, comprising adult (≥18 years) HIV-infected persons who receive health care at the UNC Hospital Infectious Diseases (ID) clinic, has been described previously [8], [9]. Over 95% of the UNC ID clinic population has consented to participate in the UCHCC and non-consenting patients do not differ significantly from those who provide consent. All patients provided written informed consent, and the study was approved by the Biomedical Institutional Review Board of the University of North Carolina at Chapel Hill.

### Study population

Antiretroviral naïve HIV-infected adults who initiated HAART from April 1996 to December 2006 were included in this analysis. HAART was defined as any combination of three or more antiretroviral agents, or a combination of at least one protease inhibitor (PI) plus one non nucleoside reverse transcriptase inhibitor (NNRTI) with or without additional agents. Patients were characterized as trial participants if HAART was initiated as part of a clinical trial. Patients co-infected with HCV and/or HBV were included in the analysis. Clinical trials included NIH AIDS Clinical Trial Group (ACTG) supported or industry sponsored trials.

### Variable Specification

We defined the outcome of virologic suppression (VS) as having a plasma HIV RNA level ≤400 copies/mL at six months from the date of HAART initiation, using a window of five to nine months and selecting the plasma HIV RNA value nearest six months if more than one value occurred in this window. We considered trial participation as the factor of interest.

A joint categorization of gender (male/female) and sexual orientation (heterosexual/homosexual/bisexual) resulted in a variable with three categories 1) men who have sex with men (MSM) 2) heterosexual men and 3) women. Bisexual men were placed in the MSM category. Additional variables included insurance status (Medicaid/Medicare, none and private/other), distance traveled from home to the UNC Infectious Diseases (ID) clinic in miles, and the duration in month's from the date of HIV diagnosis to HAART initiation.

Selected clinical laboratory values that may influence trial participation, initiation of HAART and treatment outcome including baseline CD4 cell count, plasma HIV RNA level, hemoglobin, creatinine, alanine aminotransferase [ALT], and absolute neutrophil count [ANC] were assessed. For laboratory results not available at baseline an extended window spanning 180 days before and up to 14 days after the date HAART was initiated was considered. ALT, ANC, creatinine and hemoglobin were categorized as normal or abnormal using gender appropriate normal ranges.

Treatment characteristics included the type of HAART and the date HAART was initiated. HAART was categorized as 1) a ritonavir-boosted protease inhibitor (PI) or two PIs combined with either two or three nucleoside reverse transcriptase inhibitors (NRTIs) 2) a NNRTI combined with either two or three NRTIs 3) an unboosted PI combined with either two or three NRTIs 4) a NNRTI and a PI with or without NRTIs and 5) three NRTIs. The year HAART was initiated was dichotomized to represent the differences in initial treatment regimens as the early HAART period (1996-99) and the current HAART period (2000-06) [10].

### Statistical Analyses

Baseline differences in demographic, clinical, treatment and laboratory characteristics were explored using the chi square test, t test and Wilcoxon rank sum test with 2-sided P values reported in all cases.

We estimated an unadjusted risk ratio (RR) and a 95% Confidence Interval (CI), to assess the relationship between trial participation and the risk of VS at six months after HAART initiation. Multivariable analyses were performed using Poisson regression with no offset and a robust variance estimator to provide an estimate of the risk ratio [11]–[13]. We assessed effect measure modification with interaction terms between relevant covariates. Effect measure modification was considered to be present if the coefficient estimate for the interaction term differed significantly from zero (α = 0.1). A significant interaction was noted between trial participation and the period in which HAART was initiated. Therefore all analyses were stratified by HAART period.

Confounding was evaluated by both substantive (a priori) and change in estimate criteria. We used a manual backward elimination procedure to arrive at the final model. A covariate was retained as a confounding variable if it changed the effect estimate by at least 10 percent. Two variables ‘type of HAART’ and ‘CD4 cell count’ did not change the effect estimate by ≥10% but were included in the final model based on substantive knowledge.

### Sensitivity Analyses

Our primary analysis involved a complete case analysis. HIV RNA result at the six month time point was unavailable for 33% of patients. We completed sensitivity analyses to explore the potential impact of the missing data. We conducted an extreme case analysis to obtain the upper and lower bounds of the RR [14]–[16]. For this we assumed that among the patients with missing outcome, every trial participant achieved virologic success while non-trial participants were virologic failures and vice versa. A second analysis assigned virologic failure to all or a varying fraction of missing values for trial and non-trial participants [14]–[16].

Intercooled Stata (version 9.0), Stata Corporation, (College Station, TX) was used for all analyses.

## Results

### Sample Characteristics

Of the 738 ARV naïve persons initiating HAART, 67% (n = 496) had an HIV RNA result available at six months. Results for this group (complete cases) are presented here (Table 1). The mean age of patients was 38.5 years (standard deviation 9.9), 37.3% were women, 60.1% were black, 27.4% were white, 8.3% were Hispanic and 34% initiated therapy in the context of a clinical trial. Trial participants were more likely to be MSM (42.3%), non Black (41.4%) and not have insurance (42.9%) (p<0.05). Compared to non-trial participants (14.4%) fewer trial participants (8.3%) reported injection drug use. No significant between group differences were observed in baseline HIV RNA level or CD4 cell count. Trial participants were slightly more likely to have a viral load (VL) at or close to the 6 month time point. However, the distribution around the 6 month time point was similar in that similar proportions had VL before and after 6 months in the two treatment groups (trial and non-trial).

**Table 1:** Baseline sample characteristics for study population, complete cases and comparing trial to non-trial participants restricted to complete cases.

	Study Population		Complete Cases		Non-Trial Participants		Trial Participants		p value*
	N = 738	%	N = 496	%	N = 327	%	N = 169	%	
**Demographic and Behavioral Characteristics**									
Age (years)									
<40	429	58.1	273	55	183	56	93	53.3	0.6
Gender/sexual preference									
MSM^1^/Bisexual men	252	34.2	175	35.3	101	30.9	74	43.8	0.02
Heterosexual men	260	35.2	165	33.2	114	34.9	51	30.2	
Heterosexual women	226	30.6	156	31.5	112	34.2	44	26.0	
Race									
Black	455	61.7	298	60.1	211	64.5	87	51.5	0.005
Non Black^2^	283	38.3	198	39.9	116	35.5	82	48.5	
**Access to Care Characteristics**									
Insurance Status									
Public^3^	191	26.3	126	25.9	103	31.8	23	14.1	0.001
None	276	38.1	168	34.6	96	29.7	72	44.2	
Private/Other	258	35.6	192	39.5	124	38.4	68	41.7	
Distance to ID^4^ clinic (miles)									
<50	182	24.7	130	26.3	77	23.6	53	31.4	0.06
>50	527	71.3	365	73.7	249	76.4	116	68.6	
**Clinical Characteristics**									
CD4 cells/uL									
≤200	321	56.6	257	57.6	151	54.1	106	63.4	0.1
>200–350	107	18.9	81	18.2	53	19.0	28	16.8	
>350	139	24.5	108	24.2	75	26.9	33	19.8	
Mean HIV RNA (log_10_) (sd)	4.7	(1.0)	4.9	(1.0)	4.7	(1.0)	4.8	(1.0)	0.6
Diagnosis to treatment (months)									
≤3	250	38.9	168	37.4	116	37.2	52	38.0	0.9
>3	393	61.1	281	62.6	196	62.8	85	62.0	
**Treatment Characteristics**									
HAART Initiation Year									
1996-99	266	36.0	161	32.5	124	37.9	37	21.9	0.001
2000-06	472	64.0	335	67.5	203	62.1	132	78.1	
HAART category^5^									
2 or 3 NRTI plus PI/r or 2 PI	128	17.4	99	20.0	39	11.9	60	35.5	0.001
2 or 3 NRTI plus NNRTI	288	39.0	192	38.7	132	40.4	60	35.5	
2 or 3 NRTI plus PI (unboosted)	218	29.5	134	27.0	116	35.4	18	10.7	
NNRTI/PI +/− 2 NRTI	55	7.5	40	8.1	20	6.1	20	11.8	
3 NRTI	49	6.6	31	6.3	20	6.1	11	6.5	
**Other Laboratory Parameters**									
ANC^6^ (10^9^/L)									
Normal	348	47.2	268	62.2	173	65.3	95	57.2	0.09
Abnormal	221	30.0	163	37.8	92	34.7	71	42.8	
Hemoglobin (g/dL)									
Normal	258	34.9	195	41.1	116	43.6	79	47.6	0.4
Abnormal	311	42.1	237	54.9	150	56.4	87	52.4	
Creatinine (mg/dL)									
Normal	685	93.1	462	93.2	298	91.1	164	97	0.01
Abnormal	51	6.9	34	6.8	29	8.9	5	3.0	
7ALT U/L									
Normal	451	61.1	338	81.1	204	81.3	134	80.7	0.9
Abnormal	100	13.6	79	18.9	47	18.7	32	19.3	

* p value comparing trial to non trial participants restricted to complete cases.

1 MSM = Men who have sex with Men;

2 Non Black includes Caucasian, Hispanic and Other race.

3 Public insurance =  Medicaid/Medicare;

4 ID =  University of North Carolina Infectious Disease.

5 HAART Category; NRTI – Nucleoside Reverse Transcriptase Inhibitor; PI/r – Protease Inhibitor/Ritonavir; NNRTI - Non Nucleoside Reverse Transcriptase Inhibitor.

6 ANC = Absolute Neutrophil Count.

7 ALT = Alanine amino transferase.

### Clinical Trials

Patients participated in 13 different clinical trials, nine sponsored by the AIDS Clinical Trials Group (ACTG) and four by pharmaceutical companies (Table 2)[17]–[30]. All but two of the trials were Phase III or IV. Two ACTG trials and one industry sponsored trial enrolled patients in the early period, and seven ACTG and three industry sponsored trials enrolled patients in the current HAART period.

**Table 2:** Details of Clinical Trials included in this study: Number of Patients, Study Design and Study Title.

Study	N	%	Study Design	Study Title
ACTG 384	25	14.8	Treatment, Double-Blind, Pharmacokinetics Study	Study of Protease Inhibitor and/or Non-Nucleoside Reverse Transcriptase Inhibitor With Dual Nucleosides in Initial Therapy of HIV Infection
ACTG 388	6	3.6	Treatment, Open Label, Safety Study	A Phase III Randomized, Controlled Trial of Efavirenz (EFV) or Nelfinavir (NFV) in Combination With Fixed-Dose Combination Lamivudine/Zidovudine (3TC/ZDV) and Indinavir (IDV) in HIV-Infected Subjects With Less Than or Equal to 200 CD4 Cells/mm3 or Greater Than or Equal to 80,000 HIV RNA Copies/Ml in Plasma
ACTG 5015	5	3.0	Treatment, Efficacy Study	A Phase II Exploratory Study Examining Immunologic and Virologic Indices in Two Age-Differentiated Cohorts of HIV-Infected Subjects to Explore the Basis of Accelerated HIV-Disease Progression Associated With Aging
ACTG 5073	6	3.6	Treatment, Randomized, Open Label, Uncontrolled, Parallel Assignment, Safety/Efficacy Study	A Randomized, Phase II, Open Label Study to Compare Twice Daily and Once Daily Potent Antiretroviral Therapy and to Compare Self-Administered Therapy and Therapy Administered Under Direct Observation
ACTG 5095	35	20.7	Treatment, Active Control, Safety/Efficacy Study	Phase III, Randomized, Double-Blind Comparison of Three Protease Inhibitor-Sparing Regimens for the Initial Treatment of HIV Infection
ACTG 5142	21	12.4	Treatment, Randomized, Open Label, Active Control, Parallel Assignment, Safety/Efficacy Study	A Phase III, Randomized, Open-Label Comparison of Lopinavir/Ritonavir Plus Efavirenz Versus Lopinavir/Ritonavir Plus 2 NRTIs Versus Efavirenz Plus 2 NRTIs as Initial Therapy for HIV-1 Infection
ACTG 5164	16	9.5	Diagnostic, Randomized, Open Label, Active Control, Parallel Assignment, Efficacy Study	A Phase IV Study of Antiretroviral Therapy for HIV Infected Adults Presenting With Acute Opportunistic Infections: Immediate Versus Deferred Initiation of Antiretroviral Therapy
ACTG 5175	7	4.1	Treatment, Randomized, Open Label, Active Control, Parallel Assignment, Efficacy Study	A Phase IV, Prospective, Randomized, Open-Label Evaluation of the Efficacy of Once-Daily Protease Inhibitor and Once-Daily Non-Nucleoside Reverse Transcriptase Inhibitor-Containing Therapy Combinations for Initial Treatment of HIV-1 Infected Individuals From Resource-Limited Settings (PEARLS) Trial
ACTG 5202	28	16.6	Other, Randomized, Active Control, Parallel Assignment, Safety/Efficacy Study	A Phase IIIB, Randomized Trial of Open-Label Efavirenz or Atazanavir With Ritonavir in Combination With Double-Blind Comparison of Emtricitabine/Tenofovir or Abacavir/Lamivudine in Antiretroviral-Naive Subjects
Abbott M97	7	4.1	Treatment, Randomized, Double Blind (Subject, Caregiver, Investigator, Outcomes Assessor), Parallel Assignment, Safety/Efficacy Study	Phase I/II Study of ABT-378/Ritonavir in Combination With Reverse Transcriptase Inhibitors in Antiretroviral Naive HIV-Infected Patients
Gilead 903	7	4.1	Treatment, Parallel Assignment	A Phase 3, Randomized, Double-Blind, Multicenter Study of the Treatment of Antiretroviral-Naive, HIV-1-Infected Patients Comparing Tenofovir Disoproxil Fumarate Administered in Combination With Lamivudine and Efavirenz Versus Stavudine, Lamivudine, and Efavirenz
Gilead 934	1	0.5	Treatment, Randomized, Open Label, Active Control, Parallel Assignment, Safety/Efficacy Study	Phase 3/Randomized/Open-Label Study of the Treatment of Antiretroviral-Naive HIV-1-Infected Subjects Comparing Tenofovir Disoproxil Fumarate and Emtricitabine in Combination With Efavirenz vs. Combivir (Lamivudine/Zidovudine) and Efavirenz
KLEAN	5	3.0	Treatment, Randomized, Open Label, Dose Comparison, Parallel Assignment, Safety/Efficacy Study	A Phase IIIB, Randomized, Open-Label, Multicenter Study of the Safety and Efficacy of GW433908 (700 mg BID) Plus Ritonavir (100 mg BID) Versus Lopinavir/Ritonavir (400 mg/100 mg BID) When Administered in Combination With the Abacavir/Lamivudine (600 mg/300 mg) Fixed-Dose Combination Tablet QD in Antiretroviral-Naive HIV-1 Infected Adults Over 48 Weeks

### HAART Regimens

The composition of HAART regimens significantly differed between trial and non-trial participants (p = 0.001). Trial participants were more likely to be initiated on a boosted PI regimen (60.6%) while non-trial participants were more likely to be initiated on an NNRTI based regimen (68.8%) (p = 0.001). Most patients initiating an NNRTI regimen received efavirenz (85%) while the majority of those initiating a boosted PI regimen received lopinavir/ritonavir (70.8%). Of the 134 patients who received unboosted PI regimens, 64% were initiated on nelfinavir. The most commonly used nucleoside/nucleotide backbone was lamivudine/zidovudine (49.7%), followed by lamivudine/stavudine (14.7%), tenofovir/emtricitabine or lamivudine (13.8%), and lamivudine/abacavir (7%).

**Figure 1:**
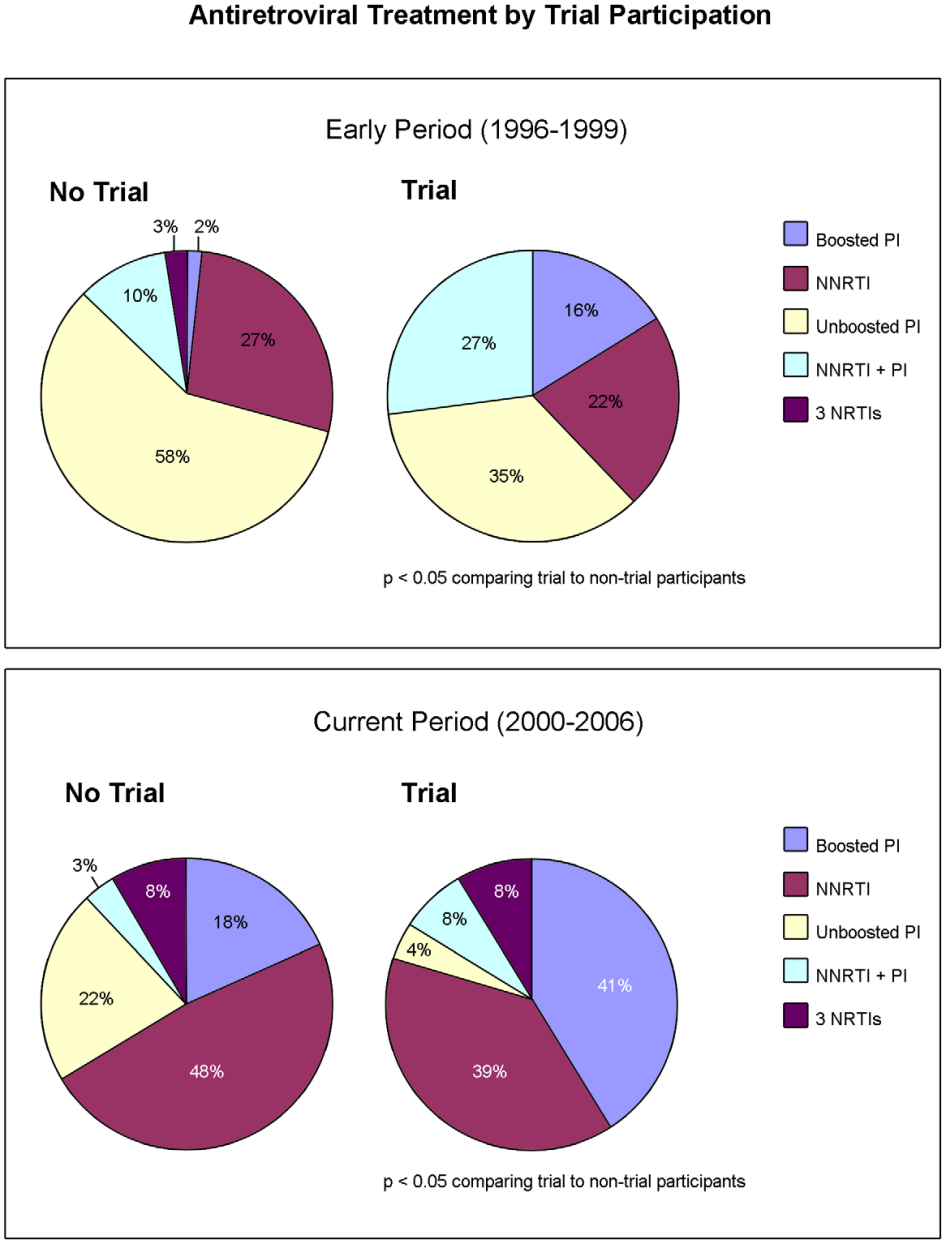
Antiretroviral treatment by trial participation.

In both the early and current periods, HAART regimens differed between trial and non-trial participants with more trial participants initiating boosted PI regimens and more non-trial participants initiating NNRTI based regimens (P<0.05) (Figure 1).

### Effect of trial participation on virologic suppression

Overall, 78% of patients achieved VS (95%CI 74, 82). Trial participants were 16% more likely to achieve VS when compared to non-trial participants (RR 1.16, 95%CI 1.06, 1.27). However the magnitude of this difference was dependent on the period in which HAART was initiated (early versus current). We thus present our results stratified by HAART period.

The effect of trial participation on VS differed by the period in which HAART was initiated (p = 0.001). In the early period, more trial participants achieved VS than non-trial participants (RR 1.42; 95%CI 1.24, 1.54). Although a difference was also observed in the current period, it was smaller and not statistically significant (RR 1.07; 95%CI 0.95, 1.19) (Table 3). After adjustment for age, distance traveled to receive care at UNC ID clinic, baseline HIV RNA levels, CD4 cell count, months from HIV diagnosis to HAART initiation, creatinine and type of HAART, trial participants remained more likely to achieve virologic suppression than non-trial participants (RR 1.33; 95% CI 1.15, 1.54) in the early period. By contrast, in the current period, virologic suppression of trial and non-trial participants was similar (RR 0.98; 95% CI 0.87, 1.11).

**Table 3:** Risk Ratios for virologic suppression by trial participation within strata of HAART period.

	Risk Ratios (95% Confidence Intervals)			
	Unadjusted		Adjusted**	
Early HAART period (1996-99)				
Non-Trial Participants	1		1	
Trial Participants	1.42	(1.24, 1.62)	1.33	(1.15, 1.54)
Current HAART period (2000-06)				
Non-Trial Participants	1		1	
Trial Participants	1.07	(0.95, 1.19)	0.98	(0.87, 1.11)

**adjusted for age, distance traveled to receive care at UNC ID clinic, baseline HIV RNA levels, CD4 cell count, months from HIV diagnosis to HAART initiation, creatinine, type of HAART.

### Missing Data and Sensitivity Analyses

The outcome of VS measured by an HIV RNA result within the specified 5-9 month window was unavailable for 242 (33%) patients. More non-trial participants (36.4%) had missing data than trial participants (24.6%) (p<0.05). Patients with missing HIV RNA results were similar to those with values in terms of age (mean age 37 vs. 39 years), race (65% vs. 60% black) and gender (29% vs. 31% female). Likewise, we found no differences in clinical characteristics (baseline HIV RNA and CD4 cell count) and laboratory parameters (ALT, ANC, hemoglobin) (all p values >0.05). However, more patients missing HIV RNA results were uninsured (45.2% vs. 34.6%) and fewer had private insurance (27.6% vs.39.5%) (p = 0.004).

**Figure 2:**
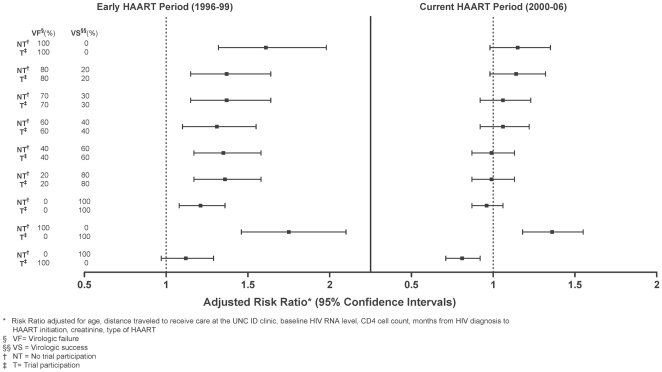
Sensitivity Analyis: Risk Ratios for viral suppression following different adjustment schema for missing data.

The proportion of patients experiencing virologic success or failure in each group or time period is not known and any assumption, including the primary analysis, has some bias. Therefore we performed a series of sensitivity analyses, in which the proportion of virologic successes and failures in patients with missing data were varied, including a missing equals failure (M = F) analysis (Figure 2). In the early period, all but one of the sensitivity analyses performed provided statistically significant risk ratios favoring trial participation. In the current period, of nine different sensitivity analyses (including the M = F analysis), trial participation was only favored in one extreme case analysis in which all trial participants with missing outcomes were considered virologic successes while non-trial participants were virologic failures (aRR 1.36, 95%CI 1.18, 1.55) (Figure 2).

## Discussion

This study is the first to examine a trial effect in HIV clinical trials by comparing virologic suppression (VS) among antiretroviral (ARV) naïve trial and non-trial participants initiating HAART. Close to two-thirds of treatment naive patients achieved virologic suppression six months after initiating HAART. The effect of trial participation varied by the period of HAART initiation. In the early HAART period (1996-99), trial participants achieved VS more commonly than non-trial participants. However, in the current HAART period (2000-06), we found no difference in VS comparing trial to non-trial participants. Our observations were supported by the sensitivity analyses.

There may be several reasons why a trial effect was readily apparent in the early HAART period. Results from cancer trials have suggested a trial effect in trials conducted before 1986, a time of rapid change for cancer care and treatments[3]. This might also be the case in our study where during the early period, there were differences in the type of HAART with more trial participants initiated on an NNRTI/PI or boosted PI combination and fewer initiated on an unboosted PI regimen (i.e., a treatment effect). However, even after controlling for differences in the type of HAART, the beneficial effect of trial participation in the early period persisted. Treatment regimens during this period were complex and associated with fairly significant side effects. Therefore, it is possible that trial participants benefited from both a care effect and a protocol effect resulting in better treatment outcomes. Furthermore, in the early period, HIV infection was associated with considerable morbidity and mortality which likely influenced patient's attitudes towards HAART. We hypothesize that, in part, the high proportion of VS experienced by trial participants resulted from a synergy between patient attitudes and care effect.

We believe our results demonstrate a true trial effect as we were able to address the challenge of identifying an optimal comparison group. Such a comparator group might comprise trial eligible subjects who declined trial participation but were similar in other baseline characteristics [3], [5]. Although we are unable to state that non-trial participants were either trial eligible or were trial eligible refusers our data suggests that our two groups were comparable. First, both trial and non-trial participants were drawn from a single clinic population suggesting similarity between groups. Second, we increased between group homogeneity by restricting our study population to include only ARV naïve subjects. Third, we controlled for treatment differences by restricting our definition of HAART and by categorizing the periods in which HAART was initiated. Finally, we examined detailed baseline demographic and clinical characteristics, as well as laboratory parameters and found no substantial differences between these two groups. Consequently it is highly likely that most of our non-trial participants were trial eligible and that some proportion of them were trial eligible refusers.

We found no strong evidence supporting a trial effect in the current HAART period. Compared to the early HAART period, the proportion of non-trial participants who achieved VS increased, but we also noted a decrease in the proportion of trial participants who achieved this milestone. The enhanced treatment response in non-trial participants may be due to noteworthy improvements in ARV therapy between the early and current HAART periods. Other cohorts examining the efficacy of triple combination therapy have reported chronological improvements in VS [10], [31]. Like other studies, we observed changes in the initial HAART regimen with a significant increase in the use of a boosted PI or NNRTI and a decline in the use of an unboosted PI [10]. The superiority of a NNRTI or boosted PI versus unboosted PI regimens in ARV naïve persons has been clearly demonstrated [19], [32]–[33]. Other improvements to ARV therapy include ease of use (dosing frequency and pill burden), better tolerability and lower toxicity. Moreover, calendar time may also be associated with other unmeasured factors such as provider experience, medication adherence and increased patient awareness about the benefits of and improvements to HAART. The period in which HAART was initiated likely acted as a surrogate for these temporal factors.

The improvements to ARV therapy in the current period may have lessened the impact of the care effect for trial participants. Additionally, in the current period HIV infection began to be regarded as a chronic but treatable infection which may have influenced patient attitudes. Thus the potential interaction between care effect and patient attitudes was likely diminished resulting in a more modest response to treatment among trial participants. Moreover, in the current period the motivation for participation in clinical trials may have changed with more patients choosing clinical trials for objective (e.g. financial) and subjective (e.g. trust in providers) reasons that we were unable to measure and which may have decreased the likelihood of success.

Our definition of VS (plasma HIV RNA ≤400 copies/ml at six months) may have limited our ability to detect a trial effect in the current period. A care effect may have needed a longer duration to become apparent in the current period as, over time, the risk for non-adherence increases and the structure of a clinical trial may improve adherence to ARV therapy and to care. Possibly, a longer outcome period might have favored trial participants and supported a trial effect. In the current period, all but one of the trials included in our study was a Phase III or later trial therefore we feel that these results are most applicable to Phase III or later trials.

Since this study was conducted at a single center these results may not be demonstrable in other settings. In our center as clinicians and clinical trial investigators overlap or are in very close contact clinical practice may be influenced by the ongoing trials. One third of our cohort was missing the outcome of VS at the six month time point. Reassuringly, patients with missing data were similar to those for whom complete data was available. Results of sensitivity analyses conducted to determine the potential influence of the missing data substantiate the results of the primary analysis and suggest that the observed effects, or lack thereof, are unlikely to be attributable to missing data. Potential sources of bias, including unmeasured confounders, could either mask or inflate a trial effect [34], [35]. We defined VS based on a single measurement, to minimize bias due to possible differences in measurement frequency between trial and non-trial participants. In our study, care setting bias and clinician selection bias appear less likely since all patients were followed at the UNC Infectious Disease Clinic and received their health care from a single group of clinicians [1], [3].

We believe this is the first study to clearly and rigorously demonstrate a trial effect in HIV clinical trials. This observation is extremely important for later lines of ARV therapy (e.g. salvage therapy) which are more complex and where the trial effect that we observed in the early HAART period might still occur due to the interplay between patient attitudes, protocol effect and care effect. Also important is the lack of strong support for a trial effect in the current period. This has significant public health implications as it demonstrates that HAART achieves comparable VS both in clinical trials and routine clinical care. These results suggest that in the current period the efficacy of HAART is no different from the effectiveness indicating that the results of clinical trials for treatment naïve HIV infected patients are generalizable to the larger population. Clinicians and public health officials can have confidence that treatment guidelines that are formulated based on clinical trials data are relevant to routine clinical care and can be extrapolated to clinical care.

Although, we found no strong evidence for a trial effect in the current period, there can be advantages to participation in clinical trials including access to newer treatments, and clinical monitoring by a dedicated team of study personnel. In keeping with other studies, we did not observe worse outcomes for trial participants in either period [36]–[40]. Therefore, we feel, a reasonable corollary is that participation in HIV treatment trials does not increase the risk of an adverse outcome. Most studies do not refute that there is a positive benefit to trial participation, though the magnitude of the benefit may differ and may depend on the type of trial [1], [41], [42]. Lastly, there is an inherent altruism involved in trial participation which may afford patients a sense of pride and self worth [43]–[46].

In summary, we demonstrated, for the first time, that participation in HIV clinical trials resulted in an improved outcome compared to clinic-based treatment (i.e. trial effect), in ARV treatment naïve patients drawn from the same population, even after controlling for multiple potential confounders. This trial effect, however, was only observed in the early HAART period and we found no strong evidence for a trial effect in HIV clinical trials in treatment naïve patients in the current HAART period. This lack of a trial effect in the current HAART period argues that for studies of combination ARV therapy in treatment naïve individuals the efficacy demonstrated in clinical trials is likely to predict the effectiveness of the therapy in broader treatment populations.
